# Characterization of cannabis strain-plant-derived extracellular vesicles as potential biomarkers

**DOI:** 10.1007/s00709-023-01870-6

**Published:** 2023-06-17

**Authors:** Ayodeji O. Ipinmoroti, Ja’kayla Turner, Elizabeth J. Bellenger, Brennetta J. Crenshaw, Junhuan Xu, Caitlin Reeves, Olufemi Ajayi, Ting Li, Qiana L. Matthews

**Affiliations:** 1 Microbiology Program, College of Science, Technology, Engineering and Mathematics, Alabama State University, Montgomery, AL 36104 USA; 2 Department of Biological Sciences, College of Science, Technology, Engineering and Mathematics, Alabama State University, Montgomery, AL 36104 USA; 3 Industrial Hemp & Specialty Crops Program, Alabama State University, Montgomery, AL 36104 USA

**Keywords:** Plant-derived extracellular vesicles, Cannabis, Fiber, Cannabidiol, Cultivar

## Abstract

The scientific interest in cannabis plants’ beneficial properties has recently sparked certain interest in the possible functional characterization of plant-derived extracellular vesicles (PDEVs). Establishing the most appropriate and efficient isolation procedure for PDEVs remains a challenge due to vast differences in the physio-structural characteristics of different plants within the same genera and species. In this study, we employed a crude but standard isolation procedure for the extraction of apoplastic wash fluid (AWF) which is known to contain the PDEVs. This method includes a detailed stepwise process of PDEV extraction from five (5) cultivars of cannabis plants, namely: Citrus (C), Henola (HA), Bialobrezenski (BZ), Southern-Sunset (SS), and Cat-Daddy (CAD). Approximately, 150 leaves were collected from each plant strain. In order to collect PDEV pellets, apoplastic wash fluid (AWF) was extracted from plants via negative pressure permeabilization and infiltration followed by high-speed differential ultracentrifugation. Particle tracking analysis of PDEVs revealed particle size distribution in the range of 20 to 200 nm from all plant strains, while PDEV total protein concentration from HA was higher than that of SS. Although HA-PDEVs’ total protein was higher than SS-PDEVs, SS-PDEVs’ RNA yield was higher than that of HA-PDEVs. Our result suggests that the cannabis plant strains contain EVs, and PDEV concentration from the cannabis plant could be age or strain dependent. Overall, the results provide a guide for the selection and optimization of PDEV isolation methods for future studies.

## Introduction

The scientific interest in cannabis plants’ beneficial properties has sparked certain interest in many areas recently ranging from recreation use, medical use, and textiles and apparel industry (Blake et al. 2017; Hill and Palastro 2017; Mahmud et al. 2021; Zimniewska 2022; Zimniewska et al. 2021). There is even further interest in cell-to-cell communication in these plants in the form of extracellular vesicles (EVs) (Jones et al. 2018), a class of vesicles that are known to be released by all forms of organisms. Isolation and functional characterization of plant-derived extracellular vesicles (PDEVs) have been used to demonstrate their contents to include biomolecules, lipids, proteins, metabolites, and small RNAs (sRNA) (Urzì et al. 2021; Cui et al. 2020). PDEVs play a crucial role in the cross-kingdom exchange of molecules ranging from plant-plant to plant host-microbes or vector trafficking molecules, especially during defense response. Establishing the most appropriate and efficient isolation procedure for PDEVs remains a challenge due to vast differences in the physio-structural characteristics of different plants within the same genera and species. For these experiments, we employed a crude but standard isolation procedure for the extraction of apoplastic wash fluid (AWF), which is known to contain the PDEVs (Huang et al. 2021).

**Fig. 1:**
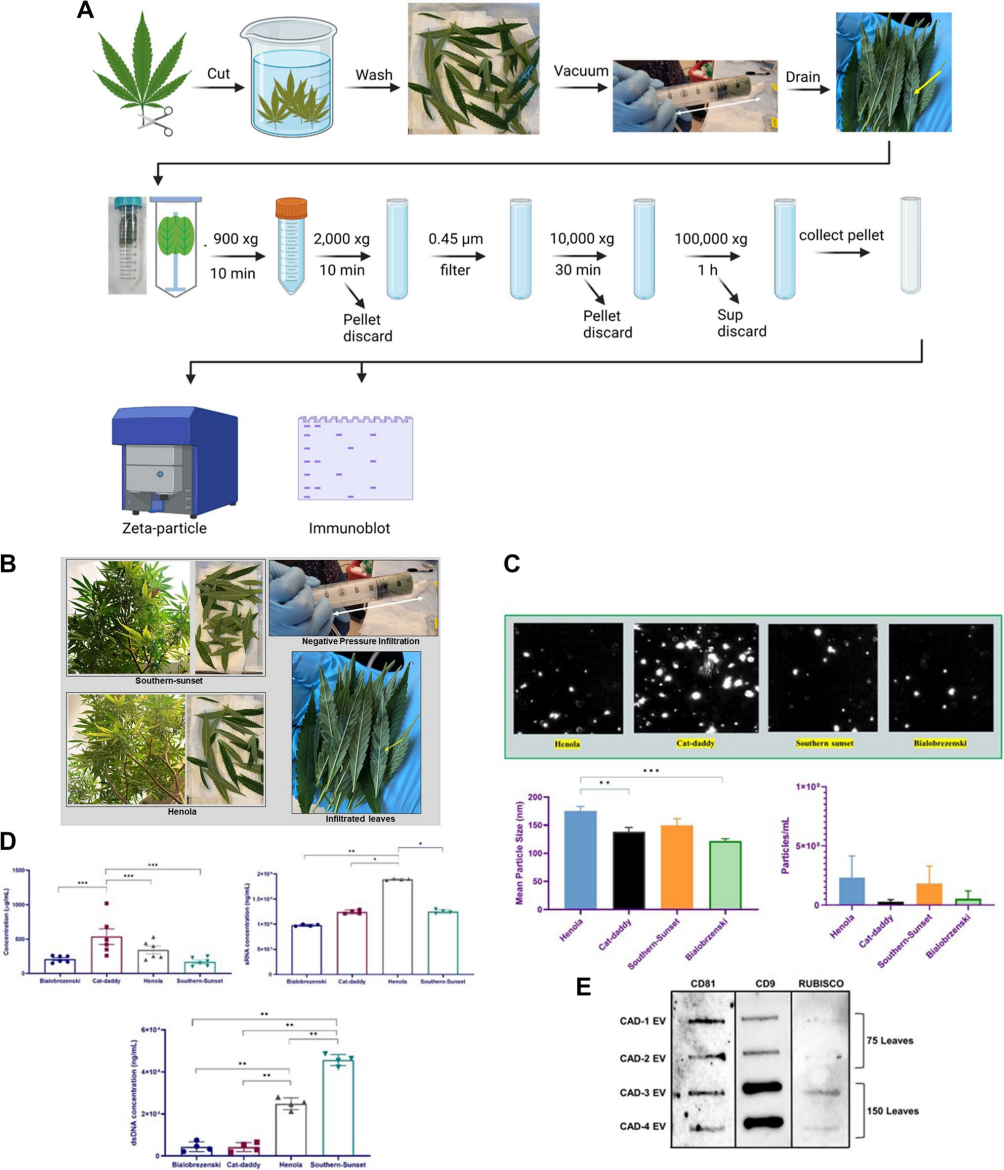
Differential expression of cannabis-derived PDEVs. **A** Diagram showing PDEV isolation workflow. **B** 75–150 leaves of each cultivar were picked and subjected to washing, negative pressure infiltration, and two rounds of high-speed ultracentrifugation. PDEVs were pelleted and quantitated for further use. **C** PDEVs particle size and concentration was quantitated via particle tracking analysis. **D** PDEVs were subjected to protein, RNA, and DNA analysis. **E** PDEVs were subjected to protein analysis via slot-blot analysis. Statistical analysis on obtained data points was performed using one-way analysis of variance (ANOVA) with Tukey post hoc analysis. Statistical significance is indicated by the mean ± standard deviation (SD) and is defined as *P* ≤ 0.05 ( ∗), *P* ≤ 0.01 (∗ ∗), and *P* ≤ 0.001 (∗ ∗ ∗)

In this study, we employed isolation of PDEVs from five (5) cultivars of cannabis plants: Citrus (C), Henola (HA), Bialobrezenski (BZ), Southern-Sunset (SS), and Southern Cat-Daddy (CAD) for these studies. The following cultivars were selected based on their usage in the production of fiber for textiles [HA, BZ] or cannabidiol (CBD) products [C, SS, CAD] products. Approximately, 75–150 leaves were collected from each plant cultivar. The plants ranged from 2 months in age to 10 months old. In brief, AWF was extracted via negative pressure permeabilization and infiltration of the plants using buffer containing [0.1 M NaCl, 30 mM (N-morpholino)ethanesulfonic acid, and 2 mM CaCl], followed by high-speed differential ultracentrifugation to collect PDEV pellets. Initially, we evaluated CAD (approximately 75 leaves) as our pilot cultivar (Fig. 1A). These PDEVs ranged in size from 10 to 1000 nm, with the average PDEVs size of all cultivars being of 121 to 175 nm (150 leaves). The mean particle size of PDEVs extracted from HA was significantly higher than that of CAD and BZ (Fig. 1B). The concentrations of PDEVs extracted from 150 leaves of each cultivar were also plentiful, ranging from 2.9 × 10^7^ to 2.3 × 10^8^ particle/mL. PDEVs were analyzed for DNA, RNA, and protein. SS PDEVs contained significantly higher DNA content in comparison to all other cultivars examined (150 leaves) (Fig. 1C and Table 1). However, HA had significantly higher sRNA content within HA-derived PDEVs isolated as compared to all other cultivars (Fig. 1C and Table 1). The PDEV isolation technique described in this study is similar to that previously described by Huang et al. (2021); however, the technique has been improved upon in this study given that we analyzed various strains of *Cannabis sativa* plant and found more tetraspanins (CD9 and CD81) including heat-shock proteins; we also employed the use of a wide range Zeta Particle Metrix analyzer for hydrodynamic particle sizes and concentration analysis. This finding is in line with HA-derived PDEVs having a large mean particle size (Fig. 1B). CAD-derived PDEVs had significantly higher protein levels in comparisons to all other cultivars examined (150 leaves) (Fig. 1C and Table 1). PDEVs from four CAD cultivars were examined for cluster of differentiations (CD) 81 and 9. CD9 was highly expressed in CAD PDEVs. All AWF from CAD plants were examined for ribulose-1,5-bisphosphate carboxylase/oxygenase (Rubisco) (75 and 150 leaves) (Fig. 1D). In addition, C, HA, BZ, SS, and CAD PDEVs all had low levels of expression of heat-shock proteins 60, 70, and 90. Rubisco protein was evaluated to rule out contamination from cytoplasmic molecules and cell debris in CAD cultivars.

**Table 1:** Chart showing total PDEV, sRNA, and DNA concentration

Cultivar name	Exosomes (mg/mL)	Volume of exosomes (mL)	RNA concentration (ng/mL)	DNA concentration (ng/mL)
Henola	343	70	10,000	45,644
Southern-Sunset	170	70	12,900	24,861
Cat-Daddy	536	100	19,029	4226
Bialobrezenski	210	100	12,950	4394

### Findings

We concluded that we could extract PDEVs from fiber and CBD cannabis plants ranging in ages from 2 to 10 months old. These findings were significant, due to the dryness of cannabis plant leaves in comparison to the leaves of *Arabidopsis* plants (Huang et al. 2021; Cai et al. 2018). Isolation and functional characterization of PDEVs have been used to demonstrate their contents to include DNA, RNA, lipids, and proteins. We found that the extraction method used was able to yield PDEVs ranging in mean size of 100–170 nm. We evaluated expression of CD9 and CD81 in the PDEVs; these tetraspanins were both expressed in CAD PDEVs. These can be markers to identify cannabis-derived PDEVs. Evaluation of several cultivars for DNA, RNA, and protein can guide further studies for potential therapeutic uses and improvements of PDEVs (Teng et al. 2021; Cho et al. 2022).
